# Rat heart T2-mapping with full coverage of the left ventricle myocardium

**DOI:** 10.1186/1532-429X-18-S1-P49

**Published:** 2016-01-27

**Authors:** Edvin Johansson, Tamsin Albery, Malin Palmér, Sven Månssonx

**Affiliations:** 1 PHB Imaging, AstraZeneca R&D, Mölndal, Sweden; 2 CVMD Cardiac Regeneration, AstraZeneca R&D, Mölndal, Sweden; 3 grid.412650.40000000406239987Department of Medical Radiation Physics, Skåne University Hospital, Lund University, Malmö, Sweden

## Background

MRI cardiac T2-mapping visualizes edema and has been used for tissue characterization in settings such as myocarditis and ischemia. Clinically it is typically performed via spin-echo based techniques, but preclinically, the high heart rates of rats and mice make such approaches sensitive to motion. Successful implementations instead employ T2-preparation followed by a segmented gradient-echo readout (Beyers *et al*. MRM 2012, Coolen *et al*. MRM 2014). It is argued here that when full coverage of the left ventricle myocardium (LVM) is needed, such techniques benefit from applying phase encoding in the slice direction as opposed to using a multi-slice approach.

## Methods

All work was approved by the local ethical committee. At day 0, 14 rats (male Clr: CD, Sprague-Dawley, ∼300 g) were subjected to sham operation (open chest surgery, non-occlusive suture in LVM). Scanning was performed at days 1, 4, and 8, under Isoflurane anaesthesia on a Bruker BioSpec 4.7T scanner equipped with a 72 mm quadrature coil. T2-preparation module: non-selective 90°-180°-90° excitation pulses with four echo times (5, 23, 41, 59 ms). Readout module: 3D gradient echo with phase encoding in the slice direction, FOV 40 × 40 mm^2^, slice thickness 1.5 mm, 128 × 126 × 8 matrix, FA 30°, TR 4.0 ms, TE 1.5 ms, bandwidth 50 kHz, 6 lines per segment synchronized to the systolic phase. The repetition time between segments was ∼2300 ms (minimum 2000 ms, but depending on the respiration rate). The acquisition time was ∼6 min. 30 s. *per* echo-time. T2 maps were calculated via the method described by Pei *et al*. MRM 2015. Regions-of-Interests (ROIs) were manually placed in the LVM and in the back muscle. ROI-level T2s were calculated as the harmonic mean of pixel-level T2s.

## Results

**Figure 1:**
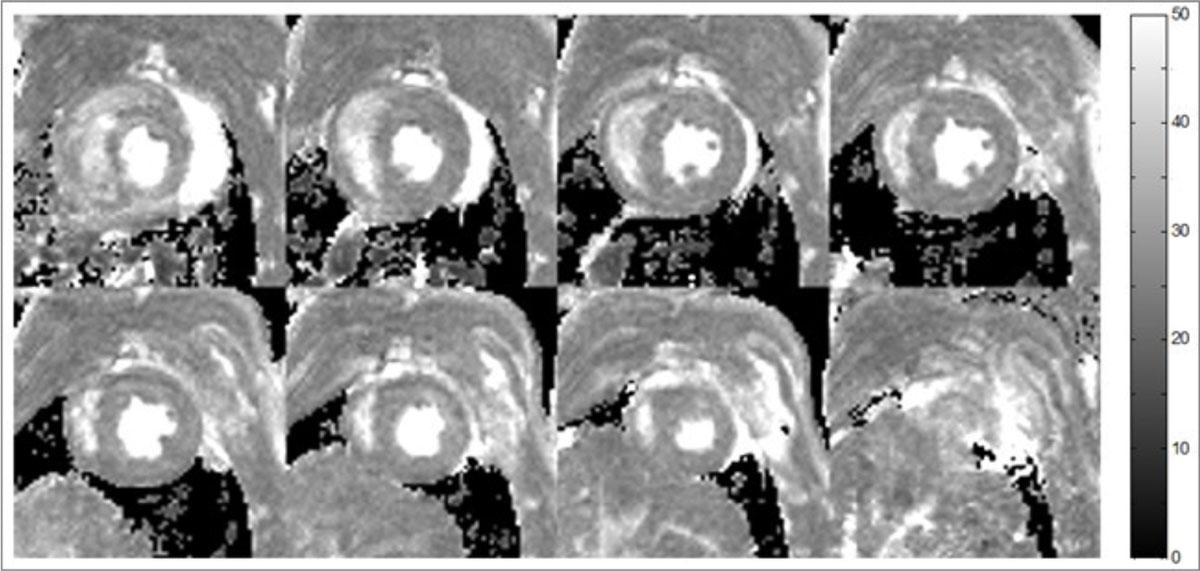
**T2 maps covering the full extent of the rat left ventricle myocardium**. Gray-scale levels express T2-values in ms. Pixels with low SNRs are shown as black.

T2 appeared homogeneous both in the LVM and in the back muscle (Figure 1). LVM T2 did not change over the eight-day period (Table 1) and remained fractionally higher than corresponding data in the back muscle at all-time points (p < 0.0001, two-sided t-test).

**Table 1:** T2 in rat left ventricle myocardium (LVM) and back muscle (mean ± standard deviation, n = 14) at days 1, 4, and 8 at 4.7T.

T2	Day 1	Day 4	Day 8
Left ventricle myocardium	29.4 ± 1.4 ms	29.1 ± 1.3 ms	29.4 ± 0.8 ms
Back muscle	26.5 ± 1.6 ms	26.1 ± 1.2 ms	26.3 ± 0.8 ms

## Conclusions

Precise T2-maps covering the full LVM were generated in sham operated rats with a signal acquisition technique earlier not used in this setting. T2 remained constant in the LV for eight days contrasting a previous report in healthy mice (Coolen *et al.* MRM 2014). When multi-slice imaging cannot be interleaved, as is the case for sequences relying on global T2 preparation, employing a phase encoding gradient in the slice direction is inherently a more efficient signal sampling strategy (Edelstein *et al.* MRM 1986). The signal-to-noise ratio (SNR) is increased by a factor √N for a given acquisition time, where N is the number of slices. Alternatively, for a fixed SNR, a potential reduction in acquisition time by a factor N can be reached. In the present study a standard volume coil was used. Further improvements in SNR or reductions in acquisition times can be obtained by combining the proposed imaging sequence with surface coils and imaging acceleration techniques.

